# Cross-talk between microtubules and the linker of nucleoskeleton complex plays a critical role in the adipogenesis of human adipose-derived stem cells

**DOI:** 10.1186/s13287-018-0836-y

**Published:** 2018-05-02

**Authors:** Yiting Yang, Rongmei Qu, Tingyu Fan, Xi Zhu, Yanting Feng, Yuchao Yang, Ting Deng, Yan Peng, Wenhua Huang, Jun Ouyang, Jingxing Dai

**Affiliations:** 1 Department of Anatomy, Guangdong Provincial Key Laboratory of Medicine and Biomechanics, School of Basic Medical Sciences, Southern Medical University, Guangzhou, 510515 China; 2 Departments of Obstetrics and Gynecology, Nanfang Hospital, Southern Medical University, Guangzhou, 510515 China

**Keywords:** Human adipose-derived stem cells (hASCs), Sad1/UNC-84 2 (SUN2), The linker of the nucleoskeleton and cytoskeleton (LINC) complex, Microtubules (MTs), Mechanotransduction

## Abstract

**Background:**

Adipose-derived stem cells (ASCs) that show multidifferentiation and anti-immune rejection capacities have been widely used in plastic and reconstructive surgery. Previous studies have indicated that mechanical and biophysical interactions between cells and their surrounding environment regulate essential processes, such as growth, survival, and differentiation, and the cytoskeleton system plays an important role in the mechanotransduction. However, the role of mechanical force in the determination of lineage fate is still unclear.

**Methods:**

Human ASCs (hASCs) were obtained from three different donors by liposuction. Adipogenesis and osteogenesis were determined by Oil Red O and Alizarin Red staining, respectively. The mRNA levels of the cytoskeleton system, PPARγ, and C/EBPα were determined by real-time polymerase chain reaction (RT-PCR). The level of cytoskeleton, PPARγ, and C/EBPα protein levels were measured by Western blotting. The morphology of the cytoskeleton system during adipogenesis was observed with confocal microscopy. hASCs were transfected with a SUN2-specific shRNA to knockdown *sun2*, and a nontargeting shRNA was used as a control.

**Results:**

We found that disrupting the physiological balance between the cytoskeleton and the linker of the nucleoskeleton and cytoskeleton (LINC) complex (especially SUN2) could impact the adipogenesis of hASCs in vitro. Microtubule (MT) depolymerization with nocodazole (which interferes with the polymerization of MTs) increased the expression of SUN2 and PPARγ, while taxol (an inhibitor of MT disassembly) showed the opposite results. Meanwhile, hASCs with *sun2* knockdown overexpressed MTs and decreased PPARγ expression, thereby inhibiting the adipogenesis. Furthermore, knockdown of *sun2* changed the structure of perinuclear MTs.

**Conclusions:**

We demonstrated the presence of cross-talk between MT and SUN2, and this cross-talk plays a critical role in the rebalance of the mechanical environment and is involved in the regulation of PPARγ transport during adipogenic differentiation of hASCs.

**Electronic supplementary material:**

The online version of this article (10.1186/s13287-018-0836-y) contains supplementary material, which is available to authorized users.

## Background

Autologous fat transfers have been successfully used for structural fat grafting in facial, lip, and hand rejuvenation and body contour improvement. One of the most interesting areas of recent research is adipose-derived stem cells (ASCs) and their potential for tissue remodeling and differentiation into specialized somatic cell types to replace damaged tissues and organs, as well as their use in cosmetic, plastic, and reconstructive surgery [1, 2]. Multiple factors determine stem cell fate, including chemical, temporal, spatial, and physical clues. However, although the regulation of stem cell differentiation by soluble factors is well characterized, the role of mechanical force in the determination of lineage fate is still unclear.

Physical cues play an important role in the determination of lineage fate in stem cells. Mechanical and biophysical interactions between cells and their surrounding environment regulate essential processes, such as growth, survival [3], and differentiation [4]. Integrin-mediated adhesion and interaction with cytoskeleton components (especially actin) are classic physical connections between cells and the extracellular matrix that regulate various aspects of cellular mechanotransduction [5]. Recent studies have suggested that the nucleus and the nuclear envelope (NE) act as mechanosensory organelles, where anchoring to the cytoskeleton through the linker of the nucleoskeleton and cytoskeleton (LINC) complex enables the transmission of mechanical force between the nucleus and the cytoskeleton outward to the external microenvironment [6, 7]. Previous studies have shown that SUN1 and SUN2 interact with lamins, other inner nuclear membrane proteins, Nesprin-1, -2, and -3, associate with actin, microtubules (MTs), and/or intermediate filaments in the cytoplasm [8]. For instance, Nesprin-1 and -2 (SYNE1, SYNE2) exist in numerous splice isoforms. The giant isoforms of Nesprin-1 and Nesprin-2 interact with actin via their N-terminal actin-binding domain, whereas other isoforms can associate with microtubule motors [9, 10].

Genetic elements of the nucleus have been shown to respond to mechanical challenges indirectly through their transduction into intermediary biochemical cascades [11], and a hypothesis has been proposed whereby applied forces might directly alter chromosomal conformation and influence the accessibility of genetic information during the binding of transcriptional enhancers or repressors [12]. However, the details of the biophysical mechanisms that control this sensitivity remain unclear.

In the process of adipogenic differentiation, human ASCs (hASCs) are transformed into lipid-filled morphologically spherical mature adipocytes. This change suggests that the transition from adult stem cells to mature adipocytes involves significant cytoskeletal reorganization or cytoskeleton remodeling [13]. Indeed, the disruption of F-actin stress fibers, which are major components of the cytoskeleton, leads to increased intracellular triacylglycerol accumulation and significantly enhanced adipogenesis [14]. Other components of the cytoskeleton have also been linked with adipogenesis, such as vimentin, which forms a cage-like structure surrounding initial lipid droplets (LDs) [15]. These studies suggest that cytoskeleton remodeling and force rebalancing is a limiting step in the morphological transition during adipogenic differentiation. However, it is not clear how remodeling of the LINC complex is regulated and initiated during adipogenesis. In this study, we investigated the changes in microtubules and the LINC complex, and we also examined its regulation by SUN2 during adipogenesis in hASCs to demonstrate the interaction between microtubules and the LINC complex and the possible mechanisms of adipogenic differentiation of ASCs.

## Methods

### Cell culture

hASCs were characterized for surface markers (Additional file 1: Figure S1). For adipogenic and osteogenic differentiation, Oil Red O and Alizarin Red staining were performed as described previously [16, 17]. hASCs between three and six passages were used in the experiments, and all experiments were repeated at least three times using hASCs from three donors. hASCs were cultured in growth medium consisting of high-glucose Dulbecco’s modified Eagle’s medium (DMEM; Gibco), 10% fetal bovine serum (FBS; Gibco), and 1% penicillin/streptomycin (Gibco).

For the adipogenesis experiments, postconfluent hASCs were cultured with adipogenic induction medium (AIM; 10% FBS, 1% penicillin/streptomycin, 10 μg/ml insulin (Sigma), 10 μM indomethacin (Sigma), 100 nM dexamethasone (Sigma), and 0.5 mM 3-isobutyl methyl-xanthine (Sigma)) for 3 days. The cells were then cultured with maintenance medium (AMM; 10% FBS, 1% penicillin/streptomycin, 10 μg/ml insulin, 10 μM indomethacin, 100 nM dexamethasone) for 1 day. The cells were then cultured with AIM to the time point for further experiments. Taxol (Selleck) was used at 10 nM and nocodazole (Sigma) was used at 0.1 μg/ml, except where noted in the figure legends. Taxol and nocodazole were added when AIM was used the first time. The cells were then cultured with AMM for 1 day. After that, the cells were cultured with AIM (without taxol and nocodazole) to the time point for further experiments (Additional file 2: Figure S2). All cells were maintained at 37 °C in a 5% CO_2_ humidified incubator.

For the osteogenesis experiments, we cultured ASCs with osteogenic induction medium (10% FBS, 1% penicillin/streptomycin, 100 nM dexamethasone, 200 μM ascorbic acid (Sigma), and 10 mM glycerol 2-phosphate (Sigma)).

### Lentivirus-mediated transduction

For knockdown of human SUN2, a SUN2-specific short hairpin RNA (shRNA: 5′-CCGGCTGCAGAAAGAAGGTGTGATTCTCGAGAATCACACCTTCTTTCTGCAGTTTTTG -3′) was packaged into a lentivirus from Genechem (Shanghai, China) (Additional file 3: Figure S4). For infection of target cells, the viral supernatants were diluted fourfold in fresh medium before transduction. Transduction was carried out in the presence of 5 μg/ml polybrene, and the medium was changed 12 h later. For the establishment of stable clones, 2 μg/ml puromycin (Santa Cruz) was added to the culture medium, and the fresh puromycin-containing medium was replaced every 3–4 days until resistant colonies were identified.

### Oil Red O staining

For Oil Red O staining, cells cultured on a cover glass were rinsed with phosphate-buffered saline (PBS), fixed with 4% paraformaldehyde (Tiengen Biological) for 10 min, and washed with 60% isopropyl alcohol (Tiengen Biological). Cells were then incubated in 2% (*w/v*) Oil Red O reagent (Cyagen) for 1 h at room temperature. Excess staining was removed by washing with 60% isopropyl alcohol, followed by several washes with distilled water. The percentage of adipogenic differentiation was determined by counting Oil Red O-positive and -negative cells (every 100 cells/field in five fields) by microscopy.

### Immunofluorescence staining

hASCs cultured on glass coverslips were fixed in 4% paraformaldehyde in PBS for 10 min at room temperature and washed with PBS. Cells were then permeabilized in 0.1% Triton X-100 in PBS at room temperature for 10 min, blocked using blocking buffer (15% goat serum in PBS) for 1 h, and subsequently incubated with primary antibodies diluted in blocking buffer overnight. In addition to the primary antibodies in the previous paragraph, antibodies against α-tubulin (1:800; mouse; clone DM1A; Abcam), lamin A (1:400; mouse; clone 133A2; Abcam), SUN2 (1:400; rabbit; Abcam), β-actin (1:500; rabbit; Sigma-Aldrich), Nesprin-1 (1:400; rabbit; clone EPR14196; Abcam), and Nesprin-3 (1:400; rabbit; Abcam) were used for immunostaining cells. Coverslips were washed with PBS for 15 min and incubated with Alexa Fluor 488-conjugated (1:500; ThermoFisher), Alexa Fluor 568-conjugated (1:500; ThermoFisher), and Alexa Fluor 633-conjugated (1:500; ThermoFisher) secondary antibodies diluted in blocking buffer at room temperature for 1 h. Coverslips were washed and mounted on to slides in 4′,6-diamidino-2-phenylindole (DAPI) mounting medium (Santa Cruz Biotechnology) and then imaged with confocal microscopy (Zeiss).

### Western blotting analysis

Cells were collected in a pH 8.0 lysis buffer (50 mM Tris-HCl, 120 mM NaCl, and 0.5% NP-40). Protein concentrations in the extracts were estimated using the Bradford method. A total of 20 μg of protein sample was loaded per lane, separated by SDS-PAGE, and then transferred to PVDF membranes. Membranes were subsequently blocked in 10% nonfat milk (Difco™ Skim Milk; BD Biosciences) in TBS with Tween 20 (TBST) for 1 h at room temperature and incubated with glyceraldehyde-3-phosphate dehydrogenase (GAPDH; 1:1000; rabbit; Cell Signaling), SUN1 (1:1000; clone EPR6554; rabbit; Abcam), SUN2 (1:1000; clone EPR6557; rabbit; Abcam), β-actin (1:500; rabbit; Sigma-Aldrich), α-tubulin (1:1000; clone DM1A; Abcam), lamin A (1:500; mouse; clone 133A2; Abcam), Nesprin-1 (1:400; clone EPR14196; rabbit; Abcam), Nesprin-2 (1:400; mouse; Abcam), Nesprin-3 (1:400), PPARγ (1:1500; rabbit; clone K.242.9; ThermoFisher), and KIF5B (1:800; rabbit; Abcam) primary antibodies at 4 °C overnight. The membranes were washed for 15 min in TBST and then incubated for 1 h with horseradish peroxidase (HRP)-conjugated secondary antibodies (1:5000; Pierce) in TBST at room temperature. After washes with TBST, the membranes were visualized by enhanced chemiluminescence (ECL) detection reagents (Applygen, China). Immunoreactive bands were detected by the ECL detection system (Protein Simple, USA), and densitometric values were analyzed with Quantity One v4.6.2 software (VersaDoc; Bio-Rad). The relative expression of each immunoreactive band was normalized to GAPDH.

### RNA extraction and quantitative real-time polymerase chain reaction analysis

Total RNA from hASCs was extracted using TRIzol reagent (Invitrogen, USA) according to instructions provided by the manufacturer. Total RNA (1 μg) was then used for reverse transcription (RT) with a commercially available kit (Revert Aid First Strand cDNA Synthesis Kit, Fermentas). Real-time polymerase chain reaction (PCR) was performed in triplicate with an ABI Step One Plus system (Applied Biosystems, USA) and a fluorescence-labeled SYBR Green/ROX qPCR Master Mix kit (Fermentas) using specific primers for α-tubulin, β-actin, SUN2, lamin A, Nesprin-2, Nesprin-3, C/EBPα, PPARγ, and GAPDH, which was used as an endogenous control. For quantification, the point of product accumulation in the early logarithmic phase of the amplification plot was defined by assigning a fluorescence threshold above the background, defined as the threshold cycle (Ct) number. Relative expression of different gene transcripts was calculated by the ΔΔCt method. The Ct of any gene of interest was normalized to the Ct of the normalizer (GAPDH). Fold changes (arbitrary units) were determined as 2 − ΔΔCt.

### Image analysis

Following immunofluorescence experiments, raw image data were analyzed using ImageJ^6^ to quantify the nuclear deformation. Simultaneously, each selection was fitted to an ellipse from which we obtained the lengths of the major and minor axes and the size of the lipid droplets. Additionally, the fluorescence intensities of Nesprin-1, Nesprin-3, and α-tubulin were measured with the ImageJ^6^ software.

### Statistical analysis

At least three independent experiments were carried out unless otherwise stated. The results are reported as the mean ± standard deviation and were analyzed using unpaired Student’s t test. The significant difference levels were set at *P < 0.05, **P < 0.01, and ***P < 0.001.

## Results

### Microtubule-based cytoskeleton reconstruction during adipogenesis

**Fig. 1:**
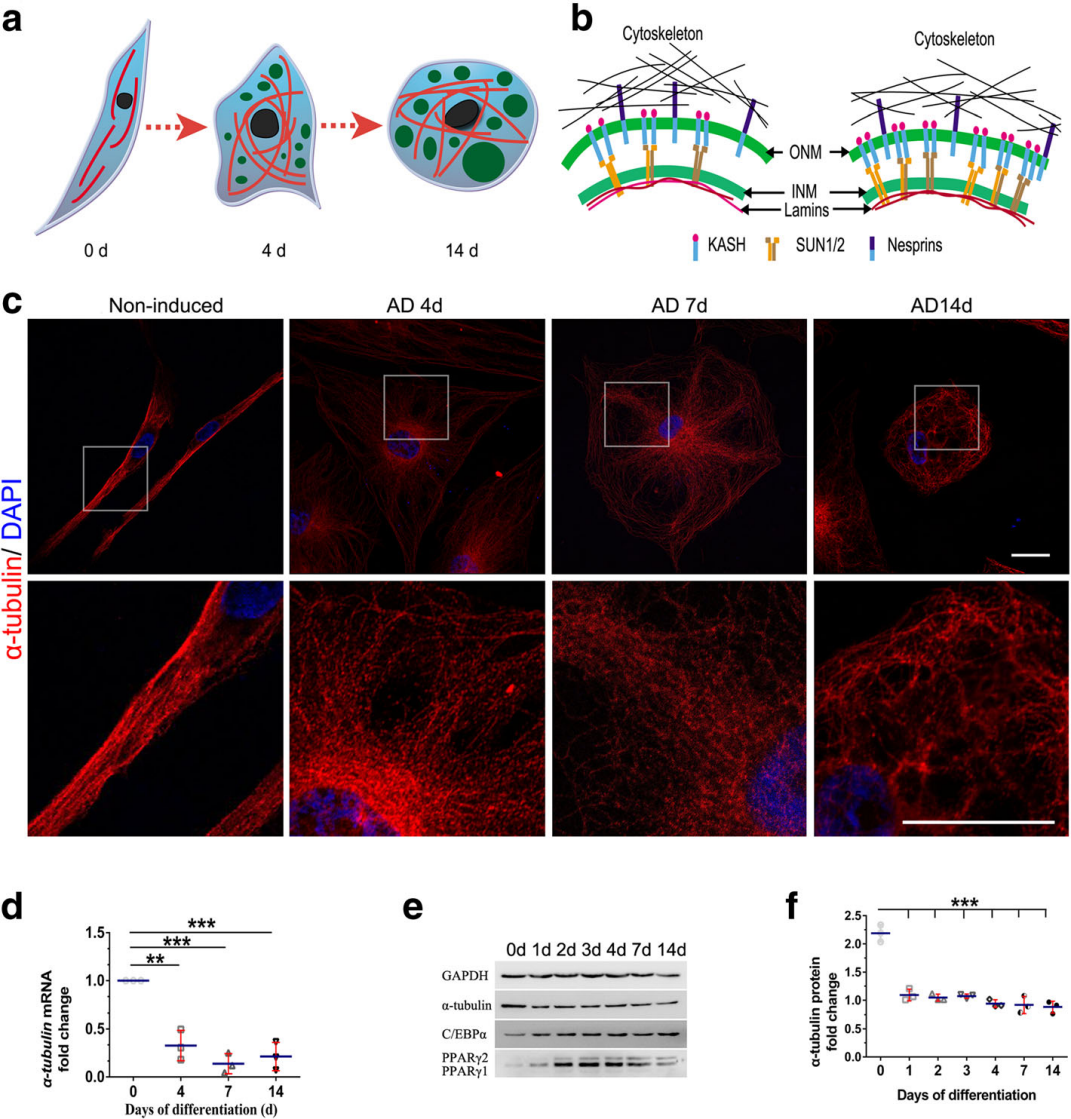
Microtubule-based cytoskeleton remodeling during adipocyte differentiation (AD) and expression of microtubules (MTs) are induced during adipogenesis. **a** A schematic drawing depicting morphological changes in the MT network during adipocyte differentiation. Lipid droplets (LDs), MTs, and nuclei are indicated by green circles, red lines, and black circles, respectively. **b** Scheme of LINC complex remodeling during adipocyte differentiation. INM, inner nuclear membrane; ONM, outer nuclear membrane. **c** hASCs at days 0, 4, 7, and 14 after adipogenic cocktail treatment were fixed, costained with anti-α-tubulin (red) and for DNA (4′,6-diamidino-2-phenylindole (DAPI); blue) and imaged using a confocal microscope for visualizing MTs and nuclei. Scale bars = 10 μm. **d** α-tubulin mRNA expression in hASCs at different days postinduction of adipogenesis was quantified by RT-qPCR; **P < 0.01, ***P < 0.001, one-way ANOVA. **e**, **f** Whole-cell lysates of differentiation-induced hASCs were analyzed by Western blotting and probed with anti-α-tubulin antibody and for the adipogenic markers C/EBPα and PPARγ. GAPDH was used to ensure equal loading. The graph shows average α-tubulin band intensities normalized to GAPDH; ***P < 0.001, one-way ANOVA

First, we analyzed cell morphology and the expression of α-tubulin at the protein level during adipogenesis of hASCs at various time points. Adipogenic differentiation was evaluated by the adipogenic markers PPARγ and C/EBPα after 14 days. In undifferentiated hASCs (day 0), microtubules were well organized in a regular array. When cells were undergoing differentiation, after the initiation of adipogenesis (day 4), the microtubule density increased around the nucleus, while peripheral microtubules became sparse and were arranged into a vacuolar structure. At the middle stage of adipocyte differentiation (day 7), microtubules remained around the nucleus at a high density and formed a cobweb-like structure. Peripheral microtubules in vacuoles fused together and gradually formed a larger gap. As cells further matured (day 14), the microtubule density around the nucleus was reduced, and microtubules translocated to the peripheral cytoplasm (Fig. 1a, c).

To detect the variations in microtubules during adipogenesis, we measured the mRNA and protein levels of α-tubulin by qRT-PCR and Western blotting analyses at various time points (days 0, 4, 7, and 14) during adipogenic differentiation. The mRNA level and also the protein level of α-tubulin was decreased during adipogenesis (Fig. 1d, e).

**Fig. 2:**
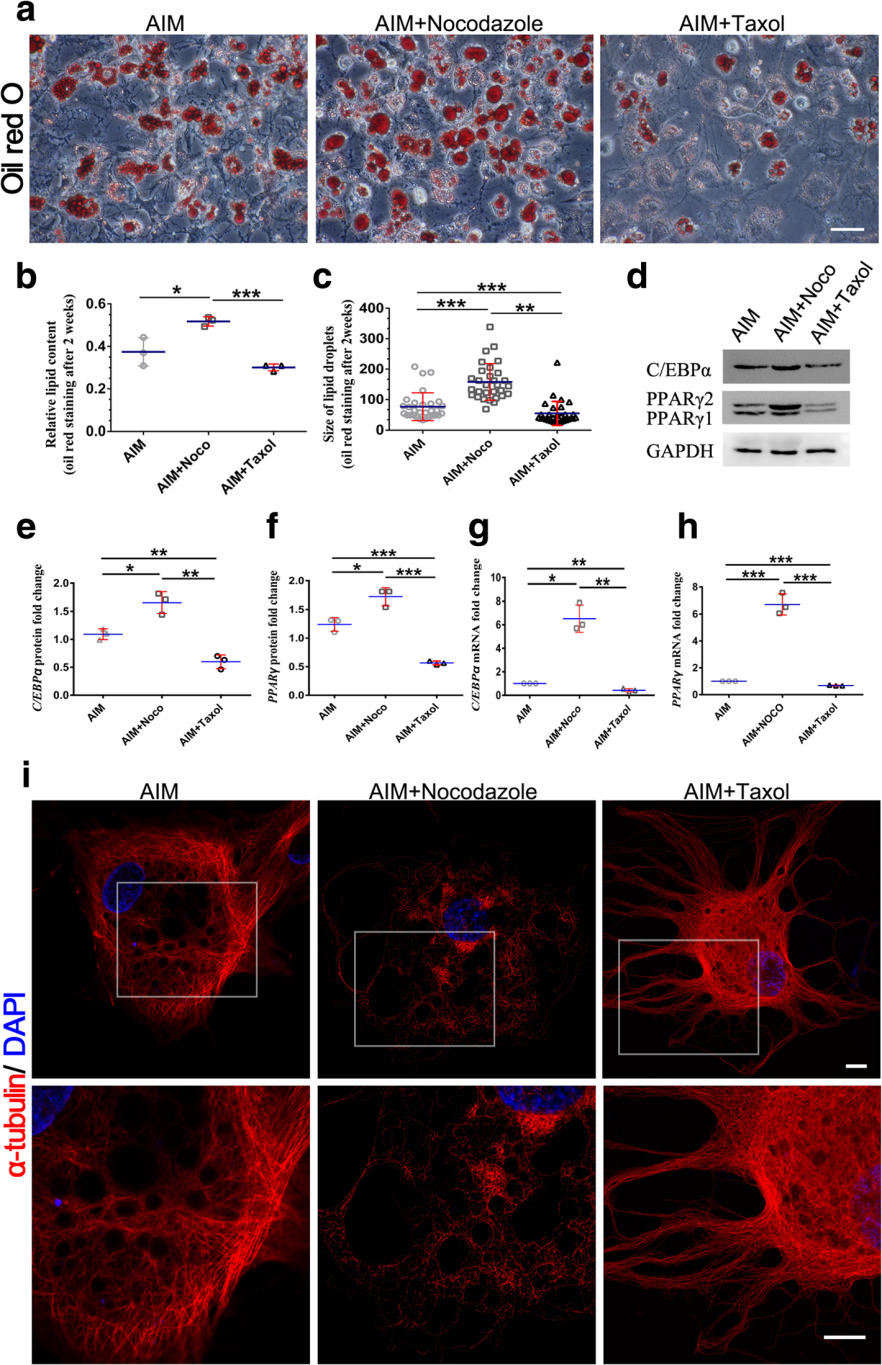
Changes in microtubule mechanical status regulates adipogenesis. **a** Oil Red O staining for triglyceride content in hASCs after 14 days of differentiation treated with nocodazole (0.1 μg/ml), taxol (10 nM), and adipogenic induction medium (AIM) only. Scale bar = 100 μm. **b** Intracellular triglyceride levels after 14 days of differentiation. Triglyceride content was normalized to total protein content and the control (AIM). One-way ANOVA was performed: *P < 0.05, ***P < 0.001. **c** Size of lipid droplets after 2 weeks of adipogenesis (*n* > 30 cells per condition). **d** The levels of C/EBPα and PPARγ were apparently increased in hASCs with nocodazole (0.1 μg/ml) (AIM+Noco) compared with the control group (AIM) at day 14 after adipogenic cocktail treatment. The taxol group (AIM+Taxol) showed the opposite results. **e**–**h** Western blotting and qPCR analysis of C/EBPα and PPARγ in hASCs from the AIM+Noco, AIM+Taxol, and AIM groups after 14 days. **i** hASCs at 14 days after adipogenic cocktail treatment or with nocodazole (0.1 μg/ml) or taxol (10 nM) were subjected to immunofluorescence staining of α-tubulin (red) and 4′,6-diamidino-2-phenylindole (DAPI; blue). Scale bars = 10 μm

To determine the effects of changes in the status of microtubule polymerization on adipogenesis, we treated hASCs with nocodazole, which interferes with microtubule polymerization and promotes microtubule disassembly, or with Taxol, which stabilizes microtubules [18]. Fourteen days after treatment with the adipogenic cocktail, microtubule density had decreased in nocodazole-treated cells, while microtubules had accumulated in the nucleus in taxol-treated cells (Fig. 2i). The total lipid content and the size of the lipid droplets increased in nocodazole-treated cells but decreased in taxol-treated cells (Fig. 2a–c).

Then, we detected PPARγ and C/EBPα at both the protein and mRNA levels after 14 days. Our research found that, compared with the control group, nocodazole-treated cells had increased levels of both PPARγ and C/EBPα; however, cells treated with taxol showed the opposite results (Fig. 2d–h). These findings indicate that the microtubule network undergoes significant changes during adipogenesis and suggest that adipogenesis can may be affected by the presence or absence of microtubule fibers (Fig. 2i). More specifically, low-stress microtubules promoted adipogenesis, while high-stress microtubules had the opposite effect. Our results suggest that changes in the abundance of microtubule fibers may regulate adipogenesis in hASCs partly by affecting the expression of PPARγ and C/EBPα.

### Actin stress fiber disruption during adipogenesis

We examined the role of actin during adipogenesis of hASCs and found that continuous F-actin fibers gradually depolymerized to monomeric G-actin after the induction of adipocyte differentiation. Meanwhile, we observed changes in the cellular localization of F-actin from localization throughout the whole cell in undifferentiated controls to localization in the peripheral cytoplasm of differentiated cells. G-actin was at the center of cells near the nuclei (Additional file 4: Figure S3).

### How does the LINC complex change during hASC adipogenesis?

**Fig. 3:**
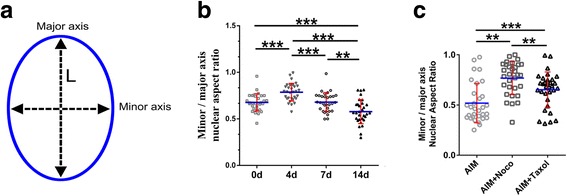
Nuclear deformation during adipocyte development. **a** Process of nuclear deformation measurement. Nuclei stained with DAPI were observed for nuclear deformation. Software (ImageJ^6^) was then used to measure the length (L) of the nuclear major or minor axis during adipogenic differentiation of hASCs at various time points. **b** The minor/major axis nuclear aspect ratio at selected time points, with more than 30 cells per time point; **P < 0.01, ***P < 0.001, one-way ANOVA. **c** The minor/major axis nuclear aspect ratio of the nocodazole (Noco) treatment group, taxol treatment group, and control group at day 14, with more than 30 cells per group; **P < 0.01, ***P < 0.001. AIM, adipogenic induction medium

Physical cues play an important role in the determination of lineage fate in stem cells [4, 19, 20]. Several studies have demonstrated that cytoskeletal organization governs nuclear deformation in response to mechanical stress, and that microtubules resist nuclear deformations in the major axis while actin resists deformations in the minor axis [21, 22]. To further elucidate the dynamic changes in the morphology around the nucleus as adipogenesis progresses in hASCs, we measured the minor/major axis nuclear aspect ratio at selected time points (Fig. 3a). The closer that this ratio is to 1, the more rounded the nuclei are (Fig. 3b, Additional file 3: Figure S4). The aspect ratio showed significant increase in the cells at day 4 and decrease at day 14 when compared with day 0 (0.6767 ± 0.01675 μm, 0.7866 ± 0.01713 μm, and 0.5778 ± 0.02320 μm at days 0, 4, and 14, respectively; 30 cells for each time point, *P* < 0.001 for day 4 and *P* < 0.001 for day 14 compared with day 0) and showed significant changes in the cells at day 4 when compared with day 14 (30 cells for each time point, *P* < 0.001). These findings indicate that during adipogenesis of hASCs the nuclei adapt to changes in the cytoskeleton by regulating their morphology [21, 22]. Furthermore, the shape of the nucleus seemed to be determined by the nucleoskeleton. In this study, we examined changes in Nesprin-1, Nesprin-3, SUN2, and lamin A in hASCs during adipogenesis.

### Nesprin-1 increased at the nuclear rim during early adipogenesis

**Fig. 4:**
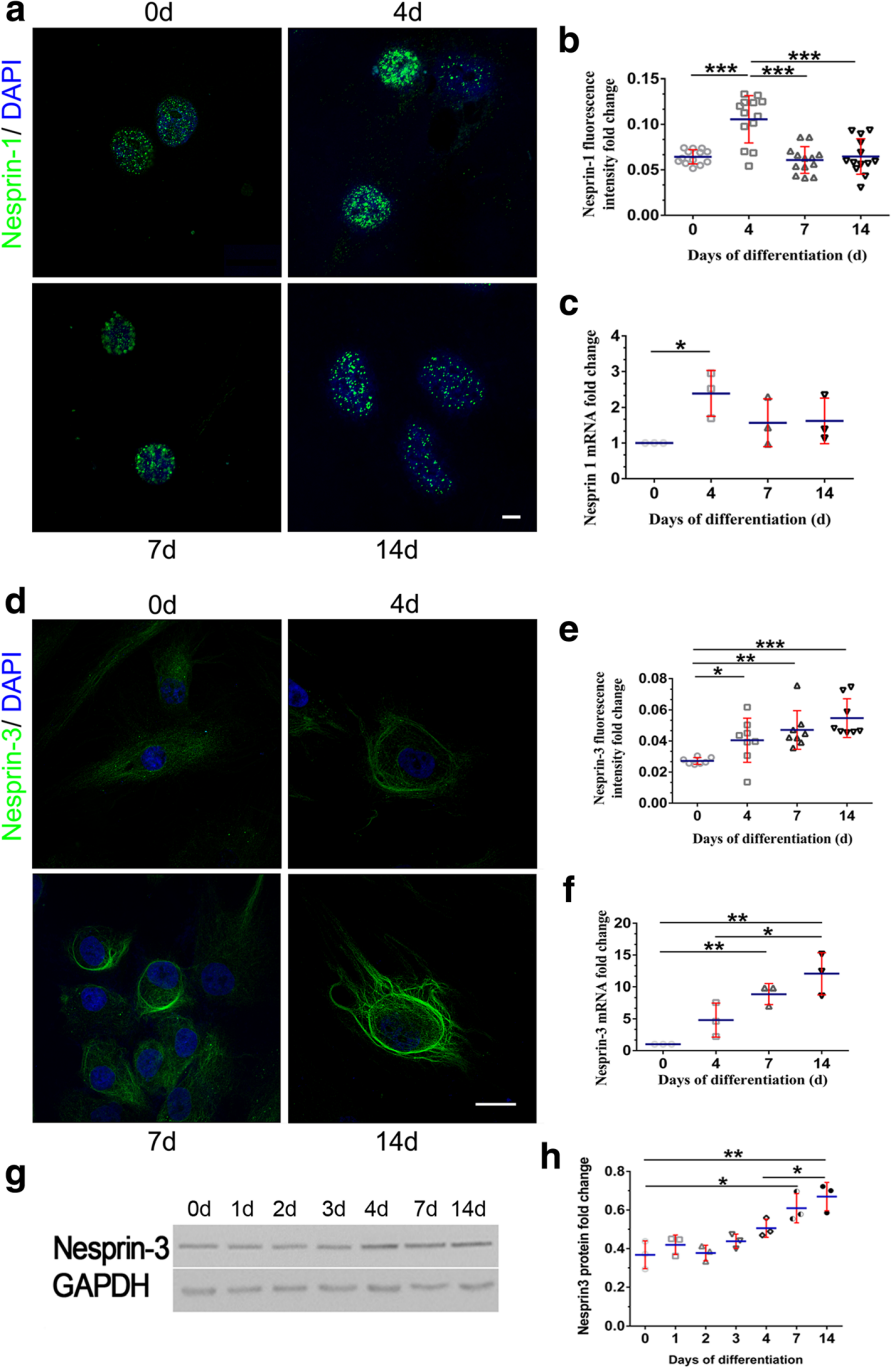
LINC changes during adipocyte differentiation. **a**, **b** hASCs at selected time points were fixed, stained with an anti-Nesprin-1 (green) antibody and/or for DNA (4′,6-diamidino-2-phenylindole (DAPI); blue), and imaged on a confocal microscope. Scale bars = 10 μm. The graph shows average Nesprin-1 (green) intensities derived from three independent differentiation experiments. *n* > 7 cells; ***P < 0.001, one-way ANOVA. **c** Nesprin-1 mRNA expression in hASCs at different days postinduction of adipogenesis was quantified by RT-qPCR; *P* > 0.05, one-way ANOVA. **d**, **e** hASCs at selected time points were fixed, stained with the anti-Nesprin-3 antibody and imaged on a confocal microscope. Scale bars = 10 μm. The graph shows average Nesprin-3 (green) intensities derived from three independent differentiation experiments. *n* > 7 cells; *P < 0.05, **P < 0.01, ***P < 0.001, one-way ANOVA. **f** Nesprin-3 mRNA expression in hASCs at different days postinduction of adipogenesis was quantified by RT-qPCR; *P < 0.05, **P < 0.01, one-way ANOVA. **g**, **h** Whole-cell lysates of differentiation-induced hASCs were submitted to Western blotting and probed with an anti-Nesprin-3 antibody. Anti-GAPDH was used to ensure equal loading. The graph shows the average Nesprin-3 band intensities normalized to glyceraldehyde-3-phosphate dehydrogenase (GAPDH) derived from three independent differentiation experiments; *P < 0.05, ** *P* < 0.01, ***P < 0.001, one-way ANOVA

Nesprin-1 forms homodimers and specifically targets the nuclear envelope through a KASH domain [23]. Experimental evidence has shown that Nesprin-1 plays a vital role in the process of mesenchymal stem cell differentiation into cardiomyocyte-like cells [24]. Other studies have shown that Nesprin-1 strongly increases in early myogenesis during human muscle development [25]. However, it is not clear how Nesprin-1 changes during adipogenesis in hASCs. To determine how Nesprin-1 responds to adipogenesis, we assessed the Nesprin-1 levels by RT-qPCR at various time points (days 0, 4, 7, and 14) during adipogenic differentiation. The mRNA level of Nesprin-1 was increased in the early stage of adipocyte differentiation on day 4 (Fig. 4c).

Next, we performed immunofluorescence staining of hASCs at various stages of adipogenic differentiation to observe the morphologic changes and to evaluate the fluorescence intensity (Fig. 4a, b). Nesprin-1 localized along the nucleus to form a dot-like structure during adipogenesis. Nesprin-1 fluorescence intensity was enhanced when adipogenesis was initiated (day 4), and then decreased in intensity as the cell further matured (days 7 and 14), which is consistent with the RT-qPCR results. The aspect ratio showed significant enhancement in the cells at day 4 compared with days 0 and 14.

### Nesprin-3 changed to a well-circumscribed circular huge ring-like structure during adipogenesis

We also investigated whether Nesprin-3 changes during adipogenesis. First, we examined the morphological dynamics in Nesprin-3 during adipogenesis. Before adipogenesis, Nesprin-3 surrounded the nucleus in a loose structure, which changed to a well-circumscribed circular huge ring-like structure that not only surrounded the nucleus but also extended to the cell periphery, with an increased fluorescence intensity after exposure to inducers of adipogenic differentiation (Fig. 4d, e). Then, we analyzed Nesperin-3 expression at the mRNA level before and after the induction of adipogenic differentiation. Nesperin-3 mRNA (Fig. 4f) gradually increased during the first week and showed a greater than 12-fold increase after 14 days, consistent with the adipogenic markers. The protein level of Nesprin-3 was increased during adipogenesis (Fig. 4g, h). Taken together, our results revealed that during adipogenesis major morphological changes occurred in Nesprin-3, and staining density and protein expression were increased.

Lamin A/C scaffolding proteins inside the NE are mechanically coupled to the cytoplasmic cytoskeletal elements via LINC complex proteins. Previous studies have shown that lamin A plays a significant role in the differentiation of both osteoblasts and adipocytes [26, 27]. In our experiment, we observed that lamin A expression peaked when adipogenesis was initiated (day 4) (Additional file 5: Figure S5), similar to other LINC proteins. All these findings demonstrate that the nucleus undergoes rapid micromechanoenvironmental changes in the early differentiation phase of hASCs.

From previous results, we found that the expression levels of the Nesprin-1 and -3 genes increased during hASC adipogenesis, and both showed upregulated expression at day 4. In morphology, the Nesprin-3 structure changed from a loose structure around the nucleus to a huge ring-like structure that extended to the periphery starting at day 4. These data indicate that Nesprin family members change their structure (especially Nesprin-3) and strength in response to adipogenesis. The initial stage of adipogenesis, at day 4, is a crucial time window.

### Upregulation of SUN2 during adipogenesis

**Fig. 5:**
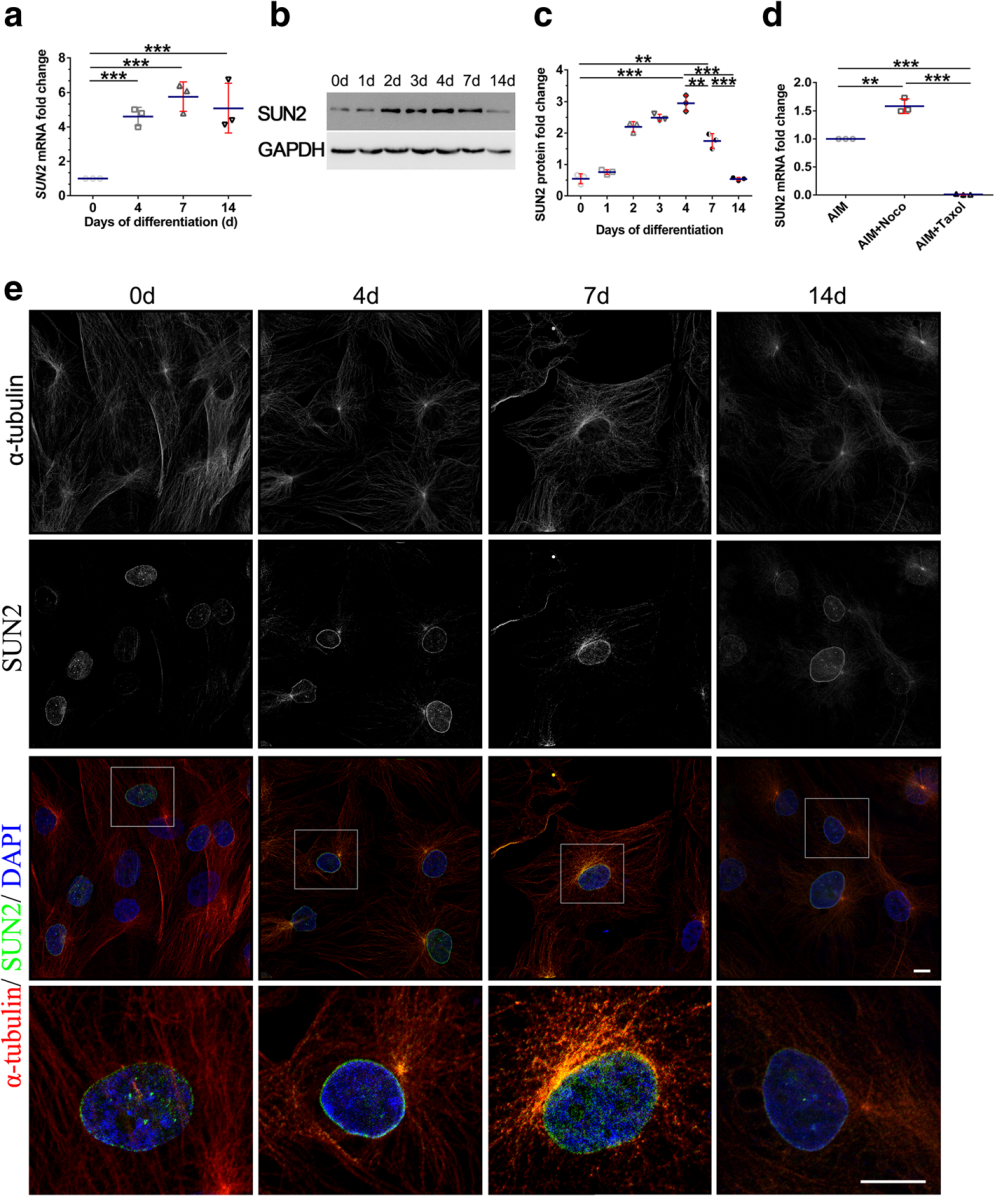
Expression of SUN2 is increased during adipogenesis. **a** SUN2 mRNA expression in hASCs at different days postinduction of adipogenesis was quantified by RT-qPCR; **P < 0.01, ***P < 0.001, one-way ANOVA. **b**, **c** Whole-cell lysates of differentiation-induced hASCs were submitted to Western blotting and probed with anti-SUN2 antibody; **P < 0.01, ***P < 0.001, one-way ANOVA. **d** SUN2 mRNA expression in hASCs with nocodazole (Noco; 0.1 μg/ml) and taxol (10 nM) after 4 days of adipogenesis was quantified by RT-qPCR; **P < 0.01, ***P < 0.001, one-way ANOVA. **e** hASCs at selected time points were fixed, stained with the anti-SUN2 antibody, and imaged on a confocal microscope. Scale bars = 20 μm. AIM, adipogenic induction medium; DAPI, 4′,6-diamidino-2-phenylindole; GAPDH, glyceraldehyde-3-phosphate dehydrogenase

To examine whether the cytoskeleton influences the nuclear response to hASC adipogenesis, we first analyzed SUN2 expression at the mRNA and protein levels during adipogenic differentiation of hASCs at various time points. As illustrated in Fig. 5a, SUN2 mRNA levels started to increase and showed significant values at day 4 and peaked at nearly sixfold by day 7, followed by a small decline until day 14 after induction. During this period, we also examined changes in SUN2 protein expression during adipogenic differentiation. SUN2 exhibited an obvious increase (fourfold) at day 2 and then gradually increased, peaking at day 4 (sixfold). SUN2 protein levels returned to their original state after 2 weeks of induction (Fig. 5b, c). Before hASCs were transferred to adipogenic media, SUN2 not only localized to the nuclear rim but also appeared at the center of the nuclei. SUN2 began to localize to the edge of the nuclei and made its first appearance outside the nucleus after adipogenesis (Fig. 5e).

Previous research has shown that cytoplasmic microtubules were mechanically coupled to the nuclear heterochromatin through proteins embedded in the nuclear envelope and that the SUN-domain played a vital role in this process [28]. These findings led us to hypothesize that extranuclear SUN2 may be associated with microtubules, especially the microtubule-organizing center (MTOC). We costained SUN2 and α-tubulin and found that they nearly overlapped. We then investigated the effect of microtubules on SUN2 gene expression (Fig. 5d) by treating cells with nocodazole and taxol after 4 days of adipogenesis. SUN2 expression increased with nocodazole (*P* < 0.01) while SUN2 expression decreased with taxol (*P* < 0.001) compared with that of day 4 hASCs cultured with only adipogenic media. We concluded that SUN2 undergoes significant changes during adipogenesis, indicating that microtubule-associated cytoskeletal changes may be involved in SUN2 regulation.

### SUN2 regulates adipogenic differentiation in hASCs by modulating PPARγ and microtubules

**Fig. 6:**
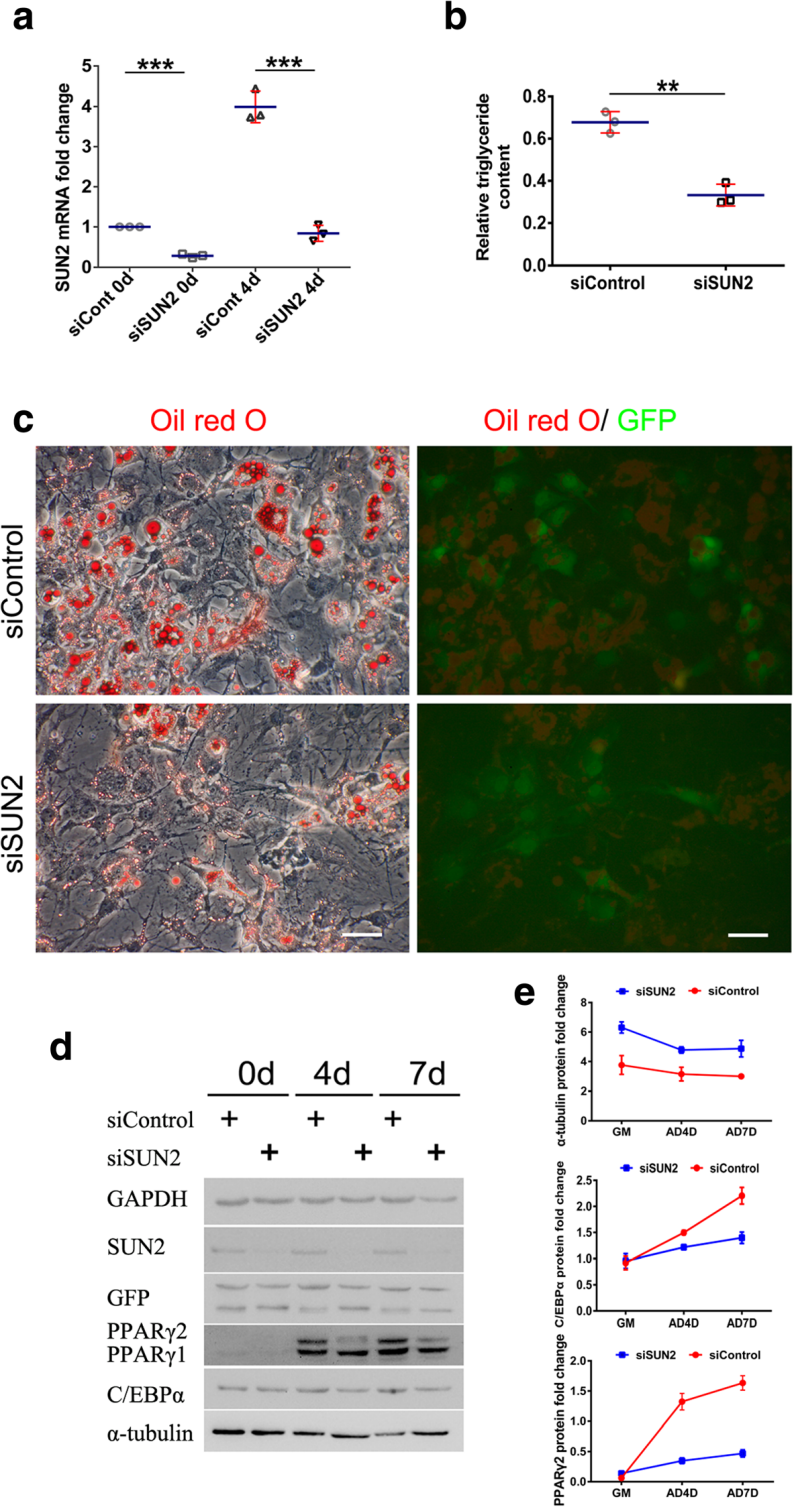
Knockdown of SUN2 inhibits adipogenic differentiation (AD) of hASCs by downregulating adipogenesis-promoting signaling pathway PPARγ and overexpressing microtubules (MTs). **a** RT-qPCR to determine knockdown efficiency in RNA samples; ***P < 0.001). **b**, **c** Oil Red O staining for triglyceride content in transfected hASCs after 7 days of differentiation. Scale bar = 100 μm. Intracellular triglyceride levels at day 7 of differentiation. Triglyceride content was normalized to total protein content and the control. Green fluorescent protein (GFP) represents transfected cells. An unpaired Student’s *t* test was performed; **P < 0.01. **d**, **e** hASC lysates of cells transfected with the indicated small interfering (si)RNAs were submitted to Western blotting and probed with an anti-SUN2 antibody and for the adipogenesis markers C/EBPα and PPARγ. Glyceraldehyde-3-phosphate dehydrogenase (GAPDH) was used to ensure equal loading and normalized α-tubulin, C/EBPα, and PPARγ2 protein levels. GM, growth medium

To determine whether SUN2 not only regulates but is also necessary for adipogenic differentiation, we transfected hASCs with a small interfering RNA (siRNA) pool against SUN2 (siSUN2) or a nontargeting control siRNA pool (siControl), and differentiation was induced 72 h post-transfection. Transfection with siSUN2 resulted in a 70% knockdown of SUN2 mRNA over the entire period of differentiation (Fig. 6a). This knockdown resulted in the formation of fewer lipid droplets compared with that of the control, as shown by Oil Red O staining (Fig. 6c) and intracellular triglyceride content (Fig. 6b).

### Knockdown of *sun2* changed the structure of perinuclear microtubules and induced microtubule upregulation during adipogenesis

**Fig. 7:**
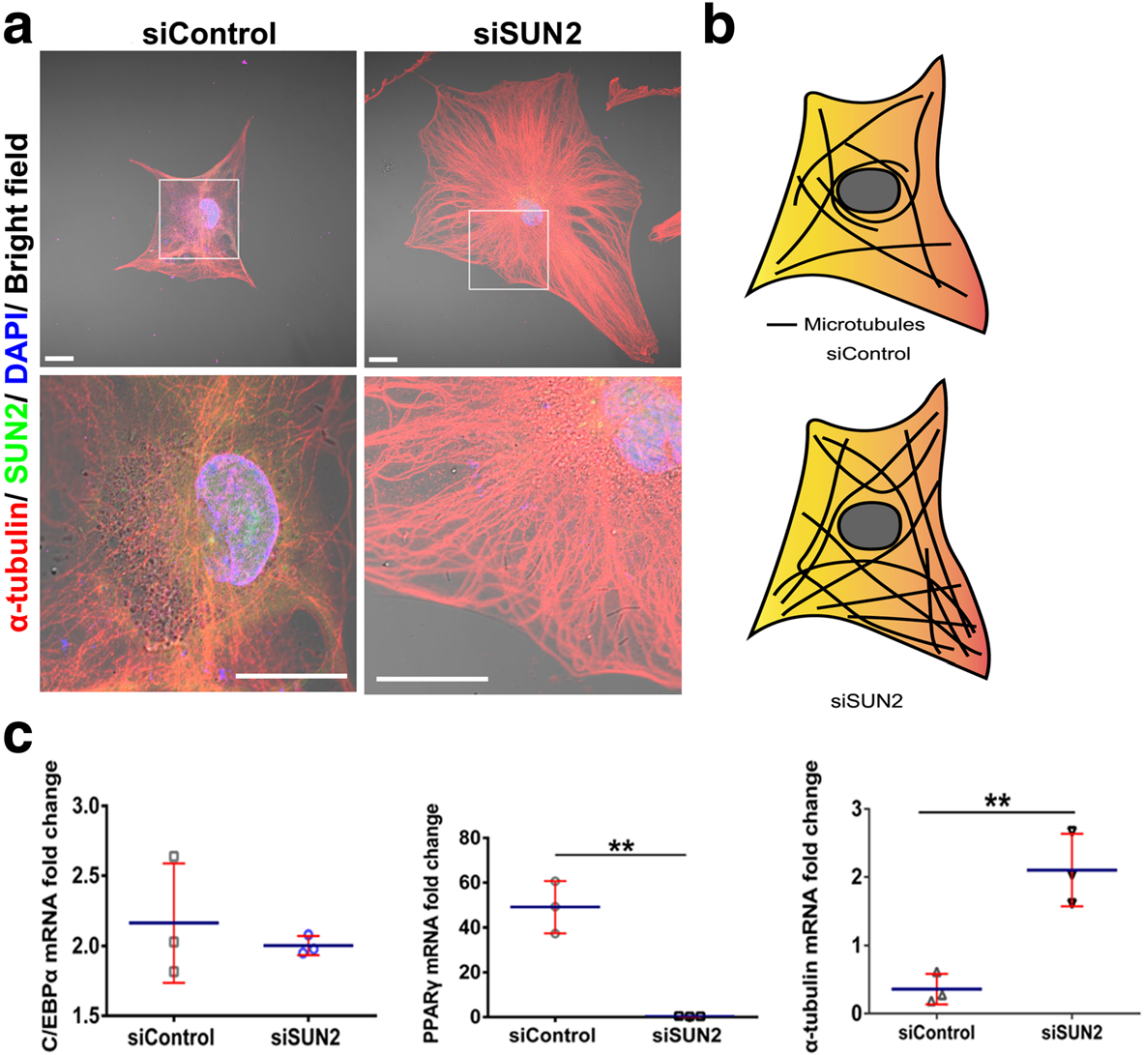
LINC complex disruption perturbs the perinuclear organization of microtubules (MTs) in hASCs. **a** Immunofluorescence analysis of *sun2*-knockdown hASCs and GFP controls. Cells were stained for α-tubulin (red), SUN2 (cherry), and DNA (4′,6-diamidino-2-phenylindole (DAPI); blue). The boxed area shows the disturbed perinuclear MT network organization. Scale bar = 20 μm. **b** Model of the effect of *sun2* loss on cytoskeletal organization in vitro. In siControl cells, MTs appeared to be shorter, sparser, and had a vacuolar structure. In siSUN2 cells, MTs maintained their integrity and rarely showed disruption. **c** RT-qPCR analysis of PPARγ, C/EBPα, and α-tubulin. Tests were performed to compare small interfering (si)Control and siSUN2-treated samples at the same time points; **P < 0.01

To determine whether there are some differences in the microtubule morphology and localization between the siSUN2 and siControl groups, we stained microtubules at an early stage (day 4) of adipocyte differentiation. On day 4, the protein expression level of α-tubulin in siSUN2-transfected cells was still greater than in the siControl treated cells (Fig. 6d, e). We observed changes in the cellular morphology of microtubules; microtubules appeared to be shorter, sparser, and had a vacuolar structure in the siControl group on day 4 (Fig. 7a, b, Additional file 6: Figure S6). In SUN2-depleted cells, microtubules maintained their integrity and rarely showed disruption. In contrast to the siControl group, in which the microtubules were around the nucleus, cells in the siSUN2 group had microtubules that formed a cap structure overlying the nuclei (Fig. 7a, b, Additional file 7: Figure S7). Our data showed that the size of siSUN2 cell maybe bigger than siControl cell although *P* > 0.05 (Additional file 8: Figure S8).

We then analyzed α-tubulin expression at the mRNA and protein levels during adipogenic differentiation of hASCs at day 4. At the cytoskeleton level, siSUN2 transfection resulted in a two- or threefold increase of α-tubulin mRNA over the 4 days of differentiation (Fig. 7c). In addition, these results suggest that LINC complex disruption (knockdown of *SUN2*) can increase the expression of microtubules, which enhances the stiffness of hASCs and perturbs the perinuclear organization of microtubules (α-tubulin) in hASCs.

### Knockdown of *sun2* induces PPARγ downregulation during adipogenesis

To investigate how SUN2 might contribute to adipogenesis, we performed Western blots and RT-qPCR analyses on day 4 of adipogenic differentiation in response to SUN2 knockdown. The mRNA levels of PPARγ and C/EBPα were assessed, with the expression of GAPDH at day 4 of adipogenic differentiation in the siControl group used to normalize the expression of the above genes. The relative expression of transcripts of the adipogenesis markers at various time points was further normalized to that of the siControl group at day 0 as control values to determine the relative fold-change in expression. PPARγ expression increased 50-fold in the siControl versus experimental cells at day 4 of adipogenic differentiation, while PPARγ expression in the siSUN2 group was at a very low level during the same period (Fig. 7c). C/EBPα levels were very similar with no significant differences between the siControl and siSUN2 cells (Fig. 7c). To evaluate the expression of adipogenesis markers and the cytoskeleton, we performed Western blot analyses at various stages (day 0, 4, 7) of adipogenic differentiation. Protein levels (Fig. 6d, e), as well as mRNA levels, of PPARγ remained low in the siSUN2 group. C/EBPα expression gradually increased during the period of a week, with the siControl group level being higher than the siSUN2 group level. These results suggested that knockdown of SUN2 suppressed adipogenesis in hASCs, especially through the inhibition of PPARγ expression and the overexpression of α-tubulin.

## Discussion

In this article, we identified a previously unknown role for microtubules and the LINC complex component SUN2, and therefore the nuclear-cytoskeletal interface, in adipogenic differentiation of hASCs. Based on our data, we demonstrated that disrupting the physiological balance between the cytoskeleton and the LINC complex (especially SUN2) could impact the adipogenesis of hASCs in vitro. By disrupting microtubules and SUN2 in hASCs, we can modulate its adipogenic differentiation, indicating that biomechanical factors (include the cytoskeleton and LINC complex) participate in stem cell adipogenesis. Meanwhile, hASCs lacking *sun2* showed overexpression of microtubules and decreased PPARγ expression, thereby inhibiting the adipogenesis. Furthermore, knockdown of *sun2* changed the structure of perinuclear microtubules.

Cytoskeleton remodeling is an essential step for the morphological transition from fibroblastic preadipocytes to lipid-filled mature adipocytes during adipocyte development. It is also a gradually remodeling process that involves a feedback mechanism to adapt to the changing mechanical environment. The cytoskeleton is a dynamic structure that primarily consists of three types of proteins: microfilaments, microtubules, and intermediate filaments [29]. Previous research has shown that F-actin and intermediate filament vimentin remodeling occurs during stem cell adipogenesis [30]. Our data also suggested that microtubules are present around the nucleus at a high density and formed a cobweb-like structure. The cytoskeleton plays important roles in regulation of nuclear shape [21, 31], and microtubules resist nuclear deformations in the major axis while microfilaments resist deformation in the minor axis [22]. Recent work also identified the nucleus and its membrane as mechanosensory organelles, where anchoring to the cytoskeleton via the LINC complex enables transmission of mechanical force between the nucleus and the cytoskeleton outward to the external microenvironment [6]. The nucleoskeleton, and associated tension inside and outside of the structure, alters the structure of the chromatin interfaces with the inner nuclear envelope and influences cell growth, division, and differentiation [32]. Cells often “soften” during adipogenesis, and hASCs need to rebalance their mechanical microenvironment to maintain the homeostasis, especially nuclear homeostasis. The importance of nuclear mechanics is observed during differentiation and development, when mechanical forces are necessary [33, 34].

LINC complex destruction can disturb the perinuclear organization of actin, vimentin, and keratin in various cell types [35–38]. Based on our data, the sudden increased density in LINC complexes (after 4 days of adipogenesis) suggests that the nucleus might reinforce itself to maintain its stability in response to mechanical environment changes, while the density of the cytoskeleton decreases. We hypothesized that the nucleoskeleton and the cytoskeleton might act as a mechanical network, similar to being on opposite sides of a seesaw, in which the strength of the nucleoskeleton falls while the strength of the cytoskeleton rises. A previous study showed that Lamin-A knockdown induced adipogenic differentiation, whereas overexpression enhanced osteogenic differentiation [27]. Interestingly, Lamin-A expression increased temporarily at the initiation of adipogenesis (day 4), although it declined in mature adipocytes (day 14) based on our data. Furthermore, our experiments suggest that SUN2 is particularly important for adipogenesis in hASCs and could not only alter microtubule perinuclear structure and enhance the microtubules but may also influence PPARγ transcription.

Microtubules have fundamental roles in many essential biological processes, such as intracellular transport which relies on the microtubule motor proteins kinesin and dynein [39, 40]. In addition, LINC also played a direct role in mRNA export through the nuclear pore complex (NPC) in mammalian cells [41]. Microtubules interface with the LINC complex through the molecular motors dynein and kinesin. Nesprin-1 and Nesprin-2 isoforms interact with the microtubule motors kinesin-1 and dynein, although whether the binding is direct is unknown [9, 42]. Mouse keratinocytes lacking SUN2 fail to correctly reorganize their microtubule cytoskeleton [43]. In conclusion, we hypothesized that microtubules and SUN2 both participate in intracellular transport of PPARγ, and the MT-KASH-SUN2 axis might play an important role in PPARγ intracellular transport. Nevertheless, the interplay among the associated factors, including the mechanotransduction system, molecular mechanisms, and the fates of the hASCs during adipogenic differentiation, is extremely complex, and there is likely to be significant cross-talk between the various factors, all of which warrants further investigation.

Several studies demonstrated that the composition of the matrix used as a scaffold for stem cells may affect the fate of the cells; a stiffer substrate induces osteogenic differentiation, whereas a less stiff substrate leads to the adipogenic lineage fate [4, 44]. These results may be mediated via changes in organization of the cytoskeleton and nuclear skeleton. Future studies are needed to demonstrate the organization of the cytoskeleton and nuclear skeleton in osteogenesis of ASCs. In future studies, we will observe the adipogenesis of ASCs under static mechanical stretching or cyclic tensile strain [45, 46] and on two-dimensional soft gel in a three-dimensional soft matrix.

## Conclusion

Taken together, our results provide new insight into the relation between the dynamics of cell shape. Our results demonstrated that cross-talk is present between microtubules and SUN2, and this cross-talk plays a critical role in rebalancing the mechanical environment and is involved in the regulation of PPARγ transport during adipogenic differentiation of hASCs.

## Additional files

Additional file 1: **Figure S1.** Identification of hASCs. (A) hASCs characterized for surface markers. (B) hASCs have the potential to differentiate towards osteocytes and adipocytes. Osteogenic differentiation: after 28 days of culture in the induction medium, hASCs stained positive for Alizarin red. Adipogenic differentiation: hASCs were subjected to adipogenic differentiation for 14 days. The formation of lipid droplets was visualized by Oil Red O staining (Scale bar = 200 μm). (TIFF 1470 kb)

Additional file 2: **Figure S2.** Time points of adipogeneic induced experiments. (TIFF 59 kb)

Additional file 3: **Figure S3.** Nuclear minor or major axis length changes. (A and B) Nuclear minor or major axis length changes at days 0, 4, 7, and 14 after adipogenic cocktail treatment. The length of the nuclear major or minor axis during adipogenic differentiation of hASCs at various time points. *n* > 30 cells; one-way ANOVA was performed: ***P < 0.001. (C and D) Nuclear minor or major axis length changes at days 0, 4, 7, and 14 after adipogenic cocktail treatment. The length of the nuclear major or minor axis after nocodazole or taxol treatment 14 days. *n* > 30 cells; **P < 0.01, ***P < 0.001. (TIFF 190 kb)

Additional file 4: **Figure S4.** Actin stress fiber disruption during adipogenesis. hASCs at days 0, 4, 7, and 14 after adipogenic cocktail treatment were fixed, costained with anti-F-actin (green) and DNA (blue) and imaged using a confocal microscope for visualizing MTs and nuclei (Scale bars = 20 μm). (TIFF 1550 kb)

Additional file 5: **Figure S5.** Lamin A changes during adipocyte differentiation. (A) hASCs at selected time points were fixed, stained with an anti-Lamin A (red) antibody and/or for DNA (blue), and imaged on a confocal microscope. Data points represent averages from three independent differentiation experiments. Scale bars = 10 μm. (B) The graph shows average Lamin A (red) intensities derived from three independent differentiation experiments. Error bars indicate SD. *P < 0.05, **P < 0.01, ***P < 0.001, one-way ANOVA. (C) Whole-cell lysate of differentiation-induced hASCs were submitted to Western blotting and probed with an anti- Lamin A antibody. Anti-GAPDH was used to ensure equal loading. (D) The graph shows the average Nesprin-3 band intensities normalized to GAPDH derived from three independent differentiation experiments. Error bars indicate SD. (TIFF 678 kb)

Additional file 6: **Figure S6.** Immunofluorescence analysis of Lentivirus-mediated transduction. For knockdown of human SUN2, we designed three different siRNAs for SUN2 from Genechem (Shanghai, China). It was proved that the *sun2* gene was knockdown in hASCs compared with the control group. (TIFF 3535 kb)

Additional file 7: **Figure S7.** LINC complex disruption perturbs the perinuclear organization of MTs in hASCs. Immunofluorescence analysis of *sun2*-knockdown hASCs and GFP control. Cells were stained for α-tubulin (red), SUN2 (cherry), and DNA (blue). The box area with disturbed perinuclear MT network organization (Scale bar = 20 μm). In siControl cells, MTs appeared to be shorter, sparser, and were changed into a vacuolar structure, while in siSUN2 cells, MTs maintained the integrity and rarely disrupted. (TIFF 1876 kb)

Additional file 8: **Figure S8.** The size of SUN2-knockdown (siSUN2) ASCs. Software (ImageJ^6^) was used to measure the size of ASCs in the siSUN2 group and the siControl group. Error bars indicate SD. *P* > 0.05, *n* = 5 cells, one-way ANOVA. (TIFF 197 kb)

## Abbreviations

AIM: Adipogenic induction medium
AMM: Adipogenic maintenance medium
ASC: Adipose-derived stem cell
C/EBPα: CCAAT/enhancer-binding protein α
DAPI: 4′,6-Diamidino-2-phenylindole
DMEM: Dulbecco’s modified Eagle’s medium
FBS: Fetal bovine serum
GAPDH: Glyceraldehyde-3-phosphate dehydrogenase
hASC: Human adipose-derived stem cell
KASH: Klarsicht, ANC-1, and Syne homology
KIF5B: Kinesin family member 5B
LD: Lipid droplet
LINC: Linker of the nucleoskeleton and cytoskeleton
MT: Microtubule
NE: Nuclear envelope
PBS: Phosphate-buffered saline
PPAR: Peroxisome proliferator-activated receptor
RT-qPCR: Real-time reverse transcription quantitative polymerase chain reaction
shRNA: Short hairpin RNA
SUN: Sad1, UNC-84
TBST: TBS with Tween 20
