# Supplementary figures and images for: Cross-talk between microtubules and the linker of nucleoskeleton complex plays a critical role in the adipogenesis of human adipose-derived stem cells
Source: Stem Cell Res Ther. 2018 May 2;9:125. doi: 10.1186/s13287-018-0836-y (PMC5930445; doi:10.1186/s13287-018-0836-y)

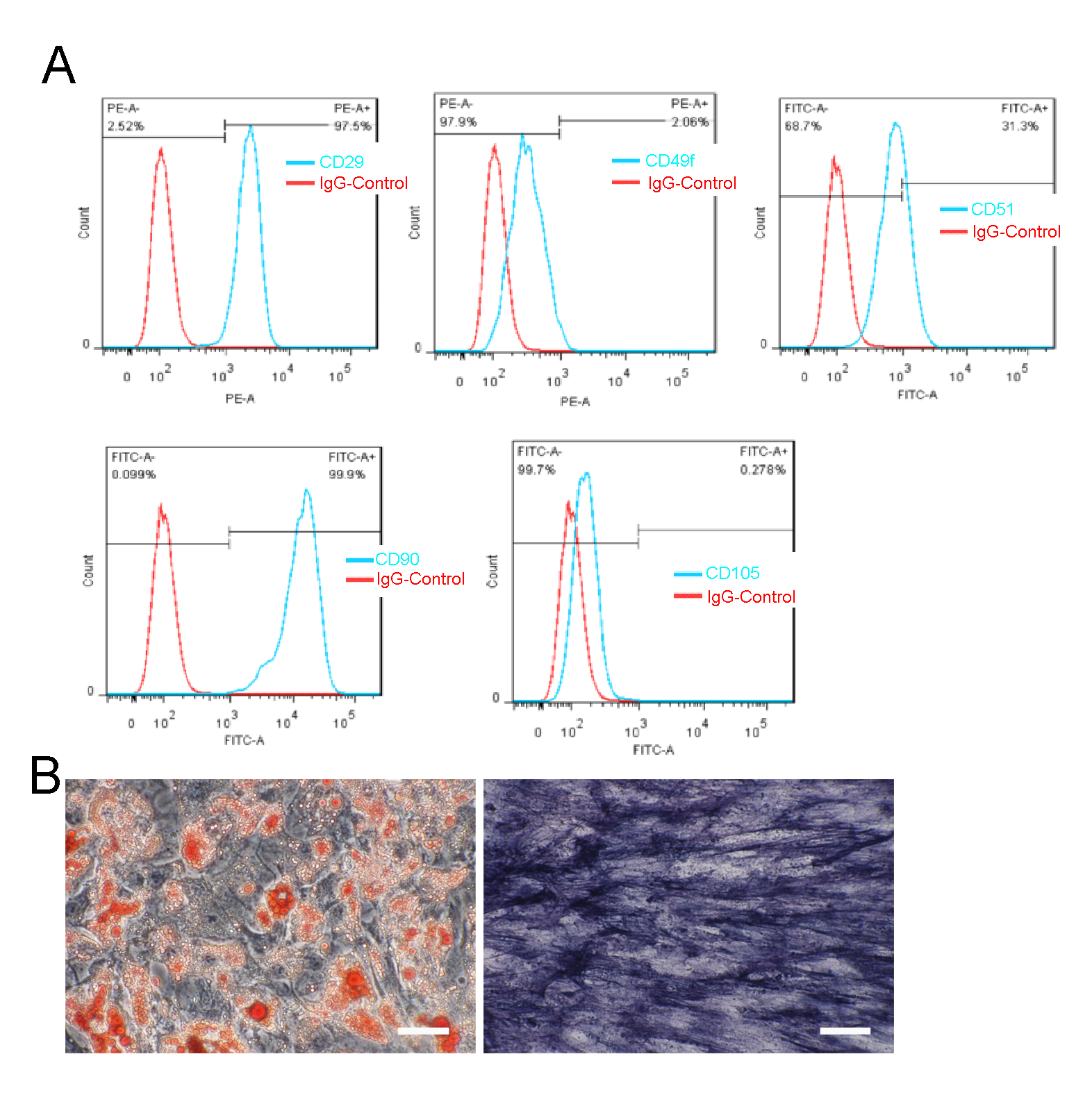

Supplement: Supplementary file 1 — Figure S1. Identification of hASCs. (A) hASCs characterized for surface markers. (B) hASCs have the potential to differentiate towards osteocytes and adipocytes. Osteogenic differentiation: after 28 days of culture in the induction medium, hASCs stained positive for Alizarin red. Adipogenic differentiation: hASCs were subjected to adipogenic differentiation for 14 days. The formation of lipid droplets was visualized by Oil Red O staining (Scale bar = 200 μm). (TIFF 1470 kb) [file 13287_2018_836_MOESM1_ESM.tif]

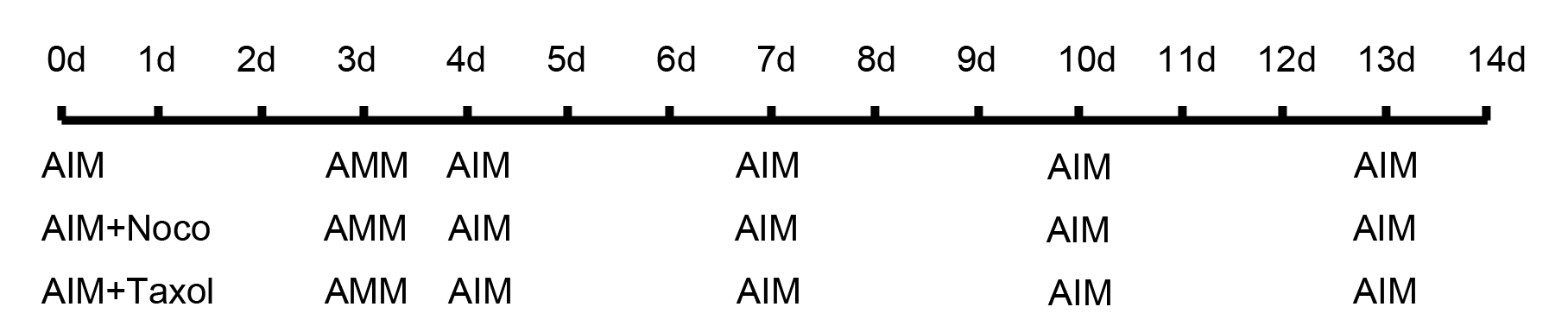

Supplement: Supplementary file 2 — Figure S2. Time points of adipogeneic induced experiments. (TIFF 59 kb) [file 13287_2018_836_MOESM2_ESM.tif]

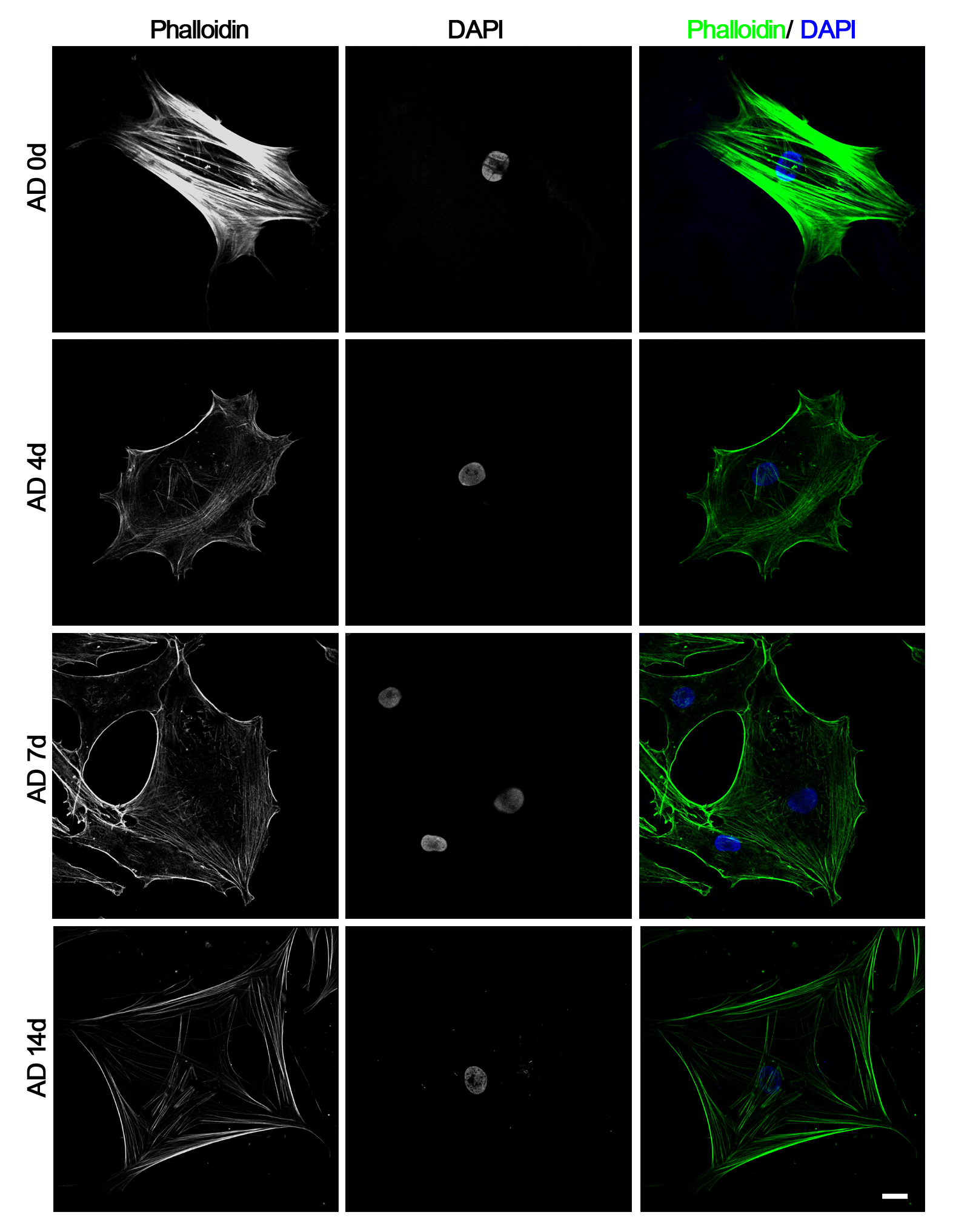

Supplement: Supplementary file 3 — Figure S3. Nuclear minor or major axis length changes. (A and B) Nuclear minor or major axis length changes at days 0, 4, 7, and 14 after adipogenic cocktail treatment. The length of the nuclear major or minor axis during adipogenic differentiation of hASCs at various time points. n > 30 cells; one-way ANOVA was performed: ***P < 0.001. (C and D) Nuclear minor or major axis length changes at days 0, 4, 7, and 14 after adipogenic cocktail treatment. The length of the nuclear major or minor axis after nocodazole or taxol treatment 14 days. n > 30 cells; **P < 0.01, ***P < 0.001. (TIFF 190 kb) [file 13287_2018_836_MOESM3_ESM.tif]

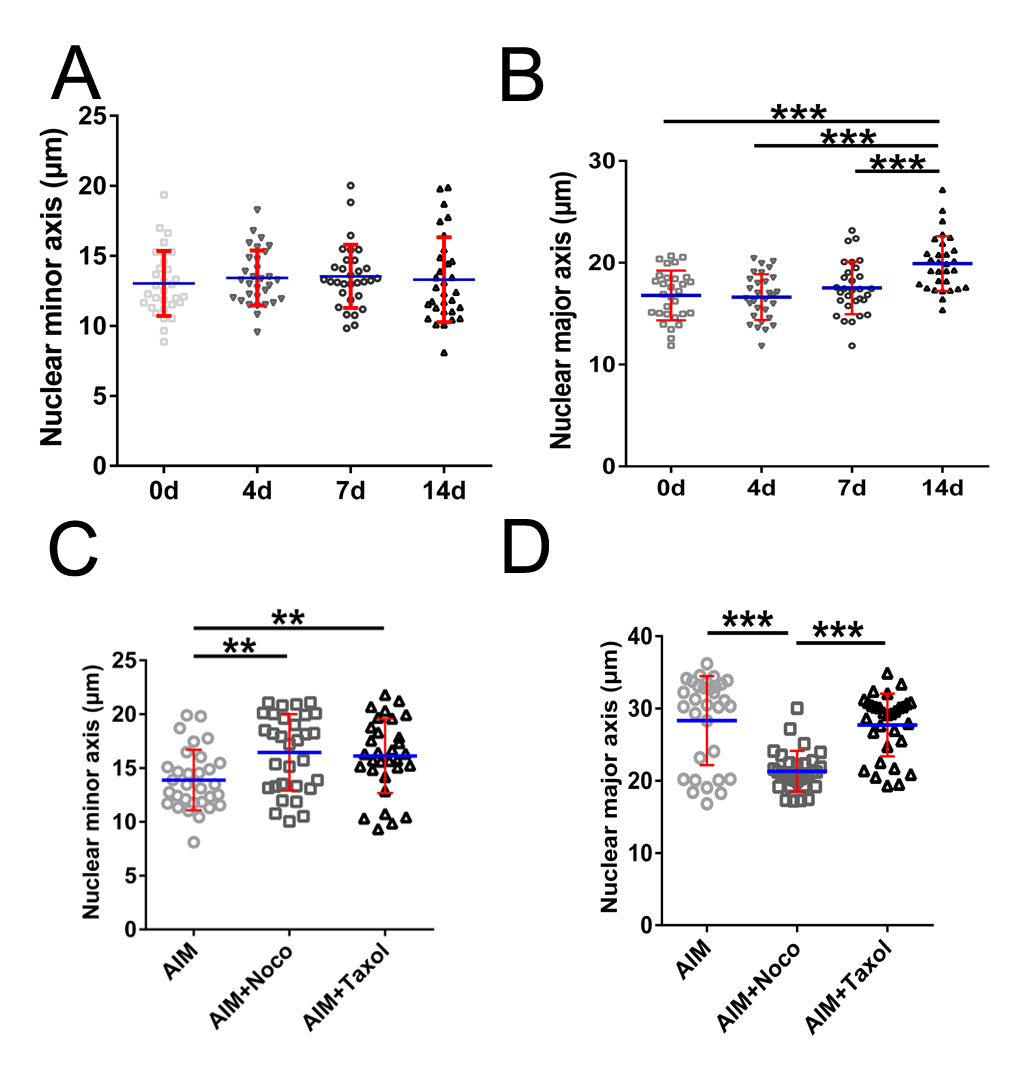

Supplement: Supplementary file 4 — Figure S4. Actin stress fiber disruption during adipogenesis. hASCs at days 0, 4, 7, and 14 after adipogenic cocktail treatment were fixed, costained with anti-F-actin (green) and DNA (blue) and imaged using a confocal microscope for visualizing MTs and nuclei (Scale bars = 20 μm). (TIFF 1550 kb) [file 13287_2018_836_MOESM4_ESM.tif]

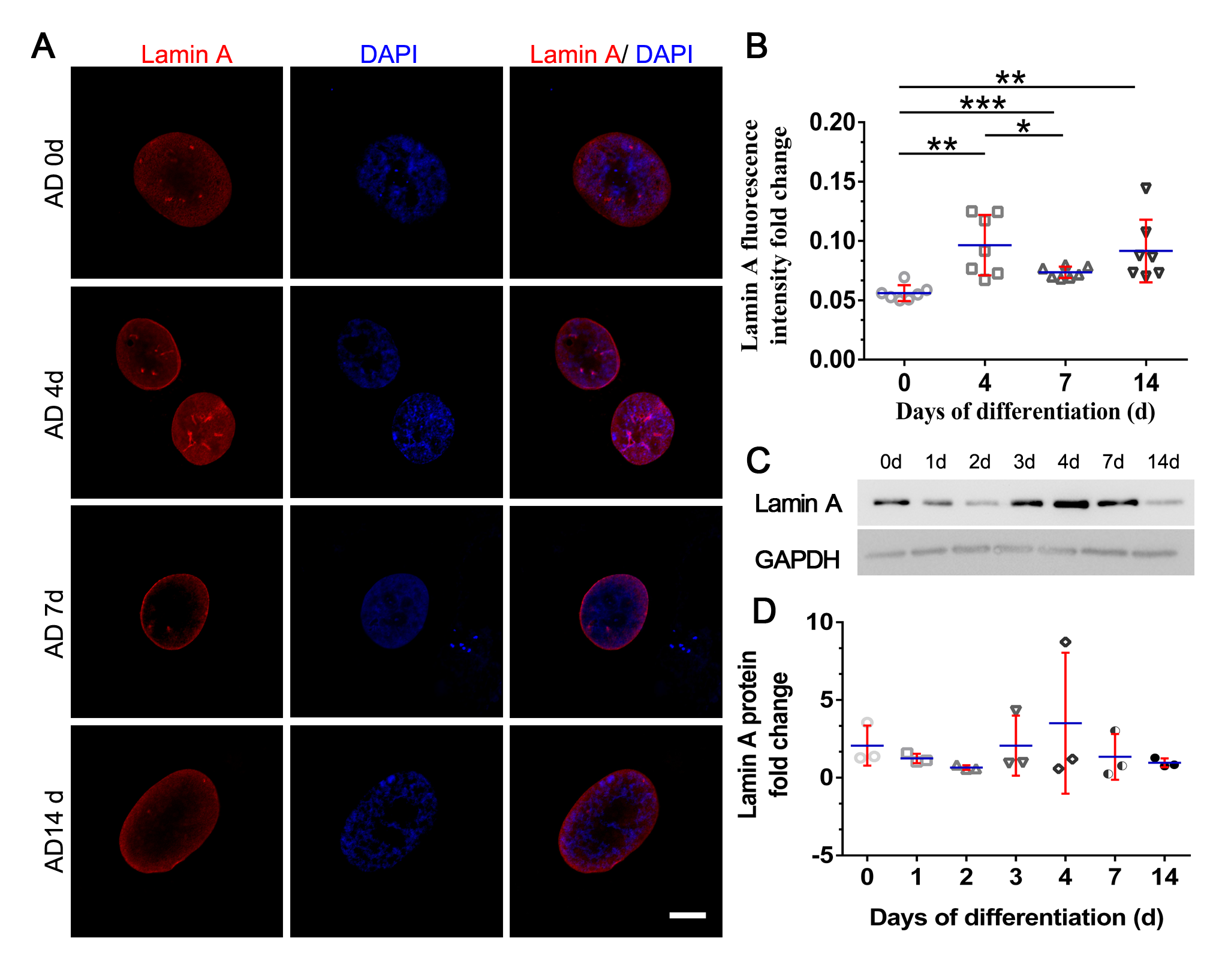

Supplement: Supplementary file 5 — Figure S5. Lamin A changes during adipocyte differentiation. (A) hASCs at selected time points were fixed, stained with an anti-Lamin A (red) antibody and/or for DNA (blue), and imaged on a confocal microscope. Data points represent averages from three independent differentiation experiments. Scale bars = 10 μm. (B) The graph shows average Lamin A (red) intensities derived from three independent differentiation experiments. Error bars indicate SD. *P < 0.05, **P < 0.01, ***P < 0.001, one-way ANOVA. (C) Whole-cell lysate of differentiation-induced hASCs were submitted to Western blotting and probed with an anti- Lamin A antibody. Anti-GAPDH was used to ensure equal loading. (D) The graph shows the average Nesprin-3 band intensities normalized to GAPDH derived from three independent differentiation experiments. Error bars indicate SD. (TIFF 678 kb) [file 13287_2018_836_MOESM5_ESM.tif]

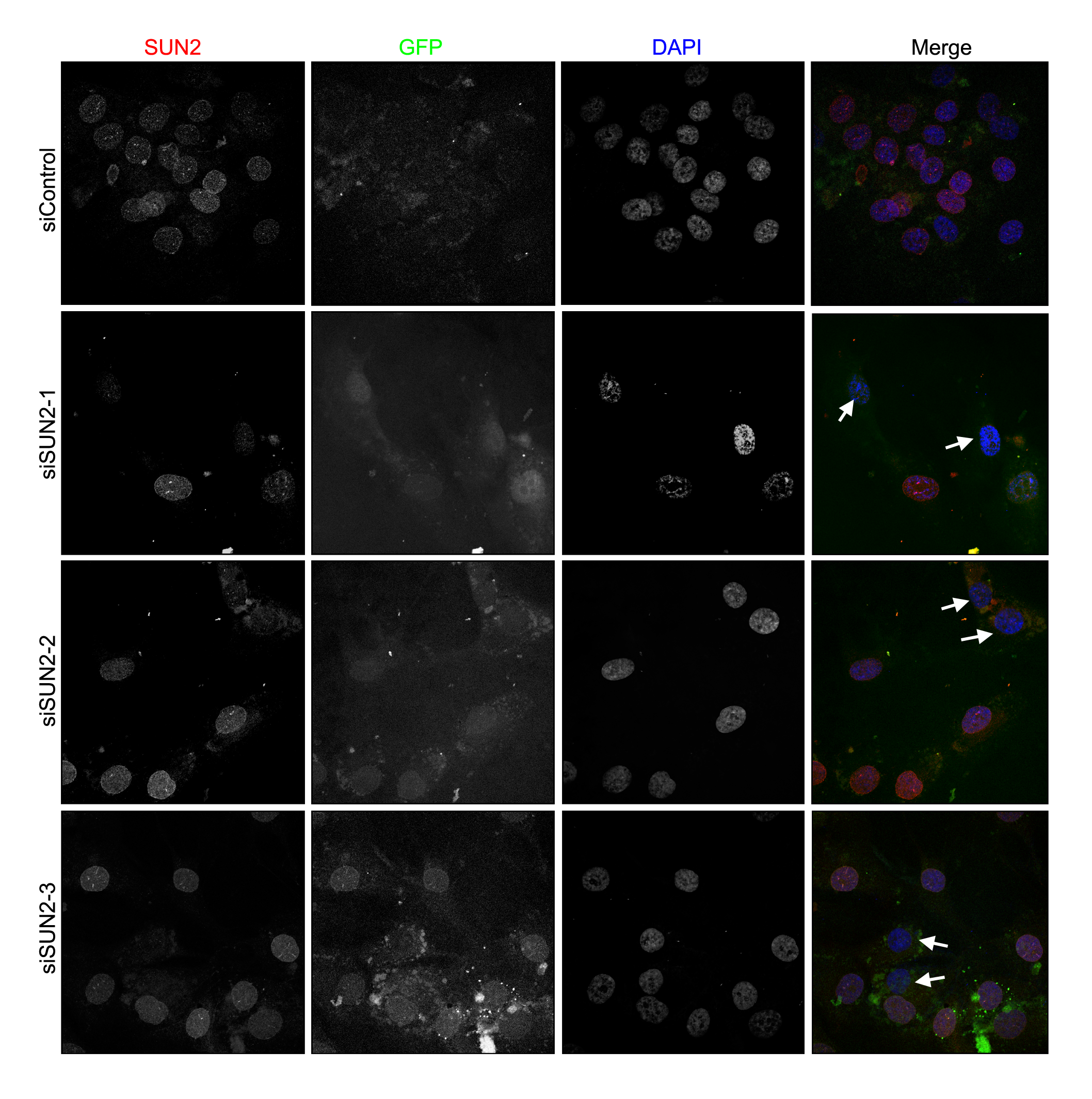

Supplement: Supplementary file 6 — Figure S6. Immunofluorescence analysis of Lentivirus-mediated transduction. For knockdown of human SUN2, we designed three different siRNAs for SUN2 from Genechem (Shanghai, China). It was proved that the sun2 gene was knockdown in hASCs compared with the control group. (TIFF 3535 kb) [file 13287_2018_836_MOESM6_ESM.tif]

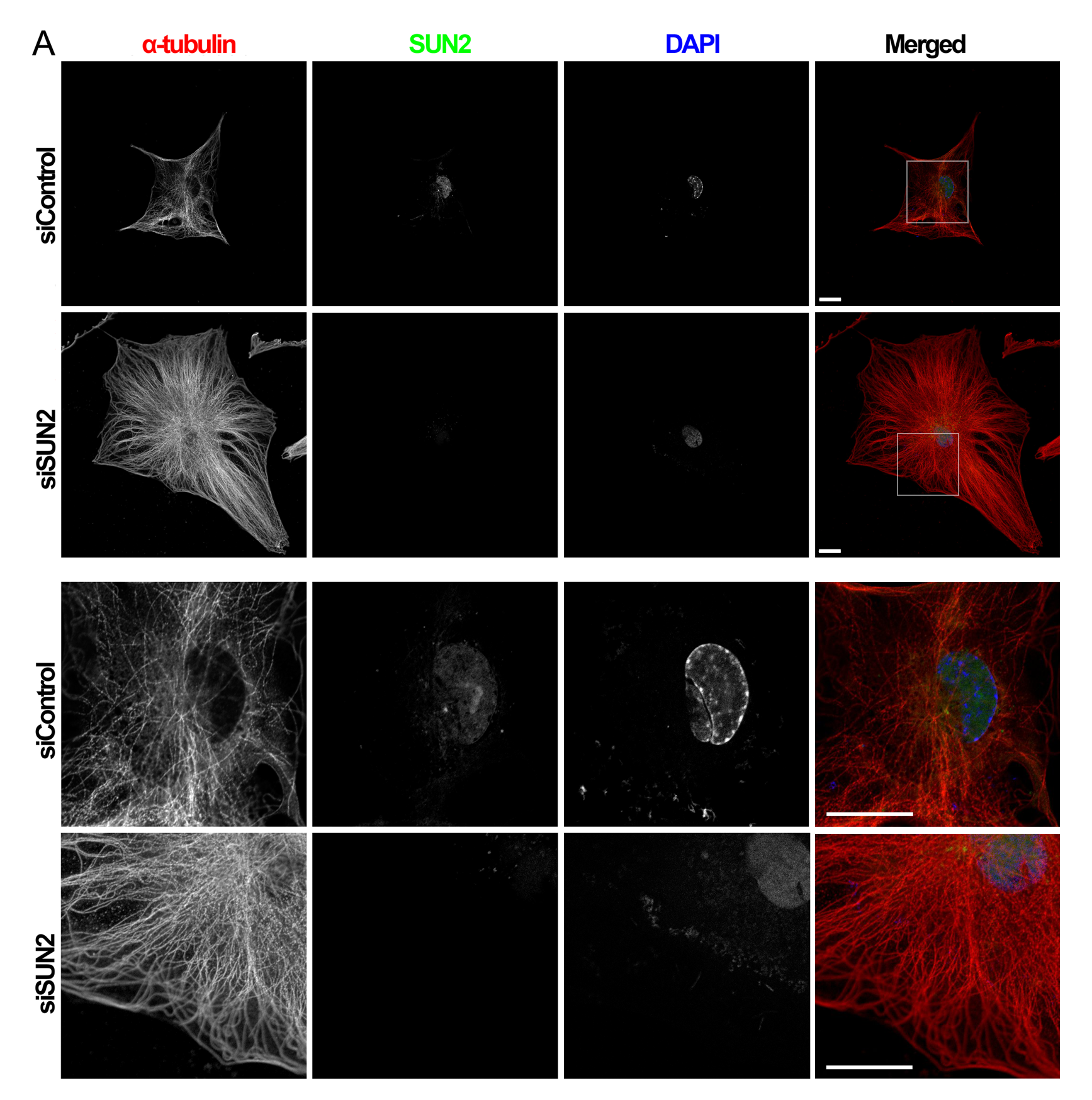

Supplement: Supplementary file 7 — Figure S7. LINC complex disruption perturbs the perinuclear organization of MTs in hASCs. Immunofluorescence analysis of sun2-knockdown hASCs and GFP control. Cells were stained for α-tubulin (red), SUN2 (cherry), and DNA (blue). The box area with disturbed perinuclear MT network organization (Scale bar = 20 μm). In siControl cells, MTs appeared to be shorter, sparser, and were changed into a vacuolar structure, while in siSUN2 cells, MTs maintained the integrity and rarely disrupted. (TIFF 1876 kb) [file 13287_2018_836_MOESM7_ESM.tif]

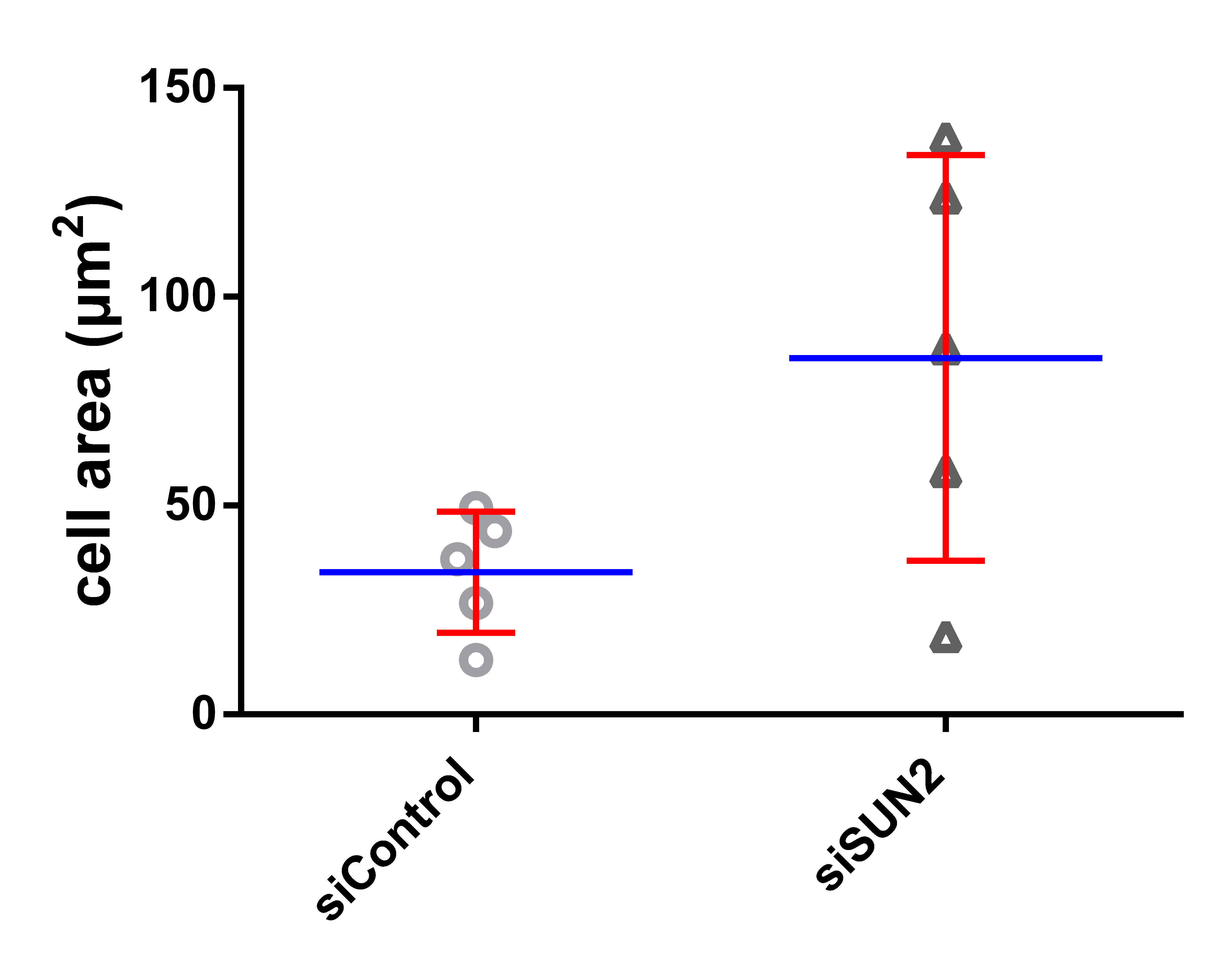

Supplement: Supplementary file 8 — Figure S8. The size of SUN2-knockdown (siSUN2) ASCs. Software (ImageJ6) was used to measure the size of ASCs in the siSUN2 group and the siControl group. Error bars indicate SD. P > 0.05, n = 5 cells, one-way ANOVA. (TIFF 197 kb) [file 13287_2018_836_MOESM8_ESM.tif]
